# School adaptation and adolescent immigrant mental health: Mediation of positive academic emotions and conduct problems

**DOI:** 10.3389/fpubh.2022.967691

**Published:** 2022-12-08

**Authors:** Lingping Xie, Weixing Zou, Hongli Wang

**Affiliations:** ^1^School of Educational Sciences, Minzu Normal University of Xingyi, Xingyi, China; ^2^College of Education for the Future, Beijing Normal University, Zhuhai, China; ^3^School of Psychology, Guizhou Normal University, Guiyang, China

**Keywords:** immigrant adolescents, school adaptation, mental health, positive academic emotions, conduct problems

## Abstract

**Introduction:**

Immigrant adolescents must adapt their physical and mental attitudes to attain healthy development due to dramatic changes in their living and learning environments after relocation. From the perspective of positive psychology, this study explored the specific influence of school adaptation on mental health among immigrant adolescents, mainly focusing on the mediating effects of positive academic emotions and conduct problems.

**Methods:**

We selected primary and secondary school students from five relocated resettlement schools in Qianxinan Buyi and Miao Autonomous Prefecture, which has the largest population of relocated people in Guizhou Province, China. Using cluster sampling, 550 relocated students in Grades 5–12 from the five schools were recruited to complete a battery of questionnaires, including the Immigrant Adolescents' School Adaptation Scale, the General Health Scale, and the Positive Academic Emotions Questionnaire, and the Adolescents' Behavioral Tendency Questionnaire. In addition, this study used the bias-corrected bootstrap method to explore the chain-mediating effect of positive academic emotions and conduct problems between school adaptation and mental health.

**Results:**

The results showed that immigrant adolescents had significant gender differences only in conduct problems. However, significant learning stage differences existed in school adaptation, mental health, positive academic emotions, and conduct problems. School adaptation, positive academic emotions, and mental health were significantly positively correlated. In contrast, conduct problems were significantly negatively correlated with mental health. School adaptation influenced mental health through the mediation effects of positive academic emotions and conduct problems. These effects contained three paths: the separate mediation effects of positive academic emotions and conduct problems and the chain mediation effect of positive academic emotions and conduct problems.

## Introduction

The term *relocated migrants* refers to individuals who move from remote places in mountainous areas to live in cities and towns. Migrants who have relocated have left their place of origin to live, study, and work in a new environment. They experience considerable changes in the environment after relocation. This change inevitably has an impact on their physical and mental health. Therefore, adapting to the new living and educational environments after the relocation and developing in the process of adaptation are practical issues that must be addressed. Due to different cultural influences, they do not adapt their behaviors and living habits immediately. Relocated young people need greater care and support in terms of academic psychology and emotional adjustment. In this study, the term *immigrant adolescents* referred to individuals aged 10–18 who have moved from regions with poor living conditions to places more suitable for survival and development (1). Academic maladjustment in the new environment may lead to negative reactions such as rebelliousness and dislike of school in young people who are relocated. Other possible adverse reactions include rebellion and academic weariness.

Empowerment theory proposes that unmet personal needs and the emergence of problems stem from the experience of exclusion and oppression of the environment. Therefore, it is necessary to enhance the ability to combat pressure from the surrounding environment and its adverse effects (2, 3). According to empowerment theory (4), immigrant adolescents experience a state of disempowerment. When undergoing relocation, they lose their original living and cultural environment, ethnic traditions, and social relations. Furthermore, after relocation, they temporarily cannot be fully integrated into the natural and social environment. As one of adolescents' main living environments, school affects adolescents' development and plays a proximal and long-lasting role in adolescents' mental health (5). Relocated adolescents are school-aged and can be empowered through favorable school resources and effective human interaction. Current research notes that the standards for school adaptation are not uniform. Existing research about circumstances pertaining to relocation has determined four aspects of relocated adolescents' school adaptation: learning, teacher-student relationship, classmate relationship, and environment. Learning adaptation refers to young people's adaptability in terms of learning attitudes and learning emotions in the face of a new school environment after relocation. Teacher-student relationship adaptation refers to how relocated students get along with teachers in a new school environment. Classmate-relational adaptation refers to peer friendships with new classmates in a new school environment. Home-learning environment adaptation refers to having an independent learning space with a better environment in the new home after relocation. Therefore, school adaptation can be used as elements of empowerment for immigrant adolescents to support adjustment to a new environment, gradually increasing psychological well-being and maintaining good long-term mental health.

Mental health has been described at different perspectives. From an adaptive perspective, it mainly includes an individual's anxiety, depression, social dysfunction, and loss of confidence (6). Studies have found that effective life adaptation is the foundation of maintaining mental health (7). In addition, it is an essential criterion for measuring mental health. Therefore, adaptability is a constituent element of mental health, and it can be measured to estimate the mental health of individuals or groups (7). A study of refugees found that the weakening of social adaptation increased internalizing behavioral problems (8). The degree of internalization of problems indicates mental health levels, and mental health outcomes of children of different ethnic cultures are influenced by acculturation. This process is promoted by the interaction of individuals with different educational levels. Cross-ethnic research has investigated cultural adaptation as an avenue to predict mental health adjustment. (9). A longitudinal study by Ratelle et al. (10) found that cognitive adaptation enhances mental health. Their study also provided indirect evidence that adaptation affects mental health. Some scholars have explored the mental health problems, such as depression and anxiety, of ethnic minorities. They found that overall ethnic minority mental health levels were lower than those of the mainstream majority population. In addition, their mental health problems were more pronounced (11, 12). Researchers have identified a relationship model connecting adaptation to mental health based on existing findings (13, 14). One finding is that, for immigrant children, maladaptation can easily lead to psychological problems due to changes in their living and learning environments.

Academic emotions refer to the various emotions that students experience after learning of academic success or failure, in classroom learning, in the process of daily homework, and during exams (15). Academic emotions can be either positive or negative. Studies have shown that improving students' learning adaptability promotes positive emotional experiences for students, and a significant positive correlation exists between learning adaptability and positive academic emotions (16, 17). These positive emotions play a supportive role during crises (18). Having been uprooted, for immigrant adolescents, the experience of positive emotions is indeed a protective factor shaping their mental health. Research has revealed that academic emotions affect students' physical and mental health (19). In addition, studies measuring emotional intelligence have found that academic emotions may affect students' mental health (20). Other studies have pointed out that anxiety affects academic stress (21). This stress is an indicator of mental health, so academic emotions can significantly predict mental health directly and indirectly (22–24). This study proposed a research hypothesis, positive academic emotions may play a mediating role between school adaptation and mental health.

Families and adolescents have similar emotional and behavioral problems (25). Emotional and behavioral problems may increase as adolescents mature (26, 27). Specific studies have shown that conduct problems are related to executive function impairment (28). In addition, low self-esteem predicts serious behavioral problems, and more conduct problems indicate diminished mental health (29). However, effective parenting is an important protective factor against children's behavioral problems (30). At the same time, emotional and behavioral problems accompany heavy psychological burdens and lower levels of mental health in adolescents (31). Conduct problems also may indicate the presence of anxiety and depression (32). It had also been shown that boys have a higher detection rate of behavior problems than girls (33), while the relative involvement of boys in behavior problems was higher than that of girls (34), and that gender could influence adolescent behavior problems (35). Under the adaptation mental health model, maladaptation may lead to conduct problem behaviors. That is, adolescents' adaptation leads to adaptation differentiation in the behavioral domain (36, 37). So, conduct problem behavior is the manifestation of adaptation differentiation, which ultimately affects mental health. Therefore, conduct problem behavior may play a mediating role between adaptation and mental health. This study presented a research hypothesis, conduct and behavior problems may play a mediating role between school adaptation and mental health.

The role of emotion in child conduct problems had been subject to growing attention in recent years (38). The emotion dysregulation was found to be a stronger predictor for the conduct problems (39). There were studies reported a negative correlation between emotion regulation and externalizing behavior problems and concluded that better emotion regulation was associated with less externalizing behaviors (40). Under the adaptive-health model, it was necessary to explore the chain-mediated mediation of academic emotion and conduct and behavior problems. Therefore, the research hypothesis was put forward, the mediating effect of academic emotions and conduct and behavior problems in school adaptation and mental health.

Poverty is associated with an increased risk of mental health problems in children (41). In addition, poverty vulnerability is an ex-ante predictor of impoverishment, distinguishing states by identifying the possibility of individuals or households succumbing to it in the future (42). Thus, combined with indicators of poverty vulnerability, relocation can effectively reduce poverty vulnerability (43, 44). For immigrant adolescents, the question remains, *What factors affect their mental health through temporarily alleviating relative poverty in living and learning conditions?* Our study aims to answer this question.

**Figure 1:**
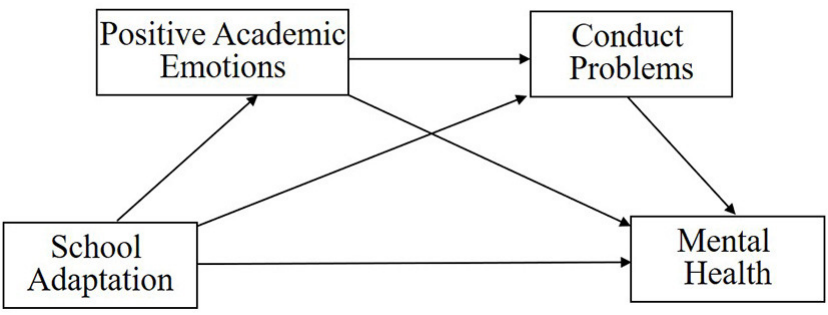
Hypothetical model: the chain mediating effect of school adaptation on mental health.

According to the theory of social exclusion (45), immigrant adolescents are inevitably overwhelmed. Being unaccustomed to the new environment, they unavoidably experience incompatibility. In the process of immigrant adolescents' adaptation, the current study aimed to understand the specific impact mechanism of school adaptation related to their mental health. This study firstly explored the relationship between school adaptation and the mental health of immigrant adolescents. Then, it examined the specific mediating mechanism of positive academic emotions and conduct problems between school adaptation and mental health. Figure 1 shows hypothetical Model 1. Therefore, on the basis of previous research, this study further explored the mechanism of school adaptation on mental health. This study not only led us to paying attention to the positive and negative effects on mental health, but also focus on the changes in school adaptation of relocated adolescents on mental health. Therefore, this study had important implications for understanding the mental health of relocated adolescents.

## Methods

### Participants

Guizhou Province is the province of China with the largest one of relocation scale and the largest overall population. Moreover, Guizhou is a multi-ethnic province. Most of the relocated population originally resided in autonomous ethnic minority autonomous areas. This study used random sampling to select primary and secondary students from five resettlement schools in Qianxinan Buyi and Miao Autonomous Prefecture, which has the largest poverty alleviation and relocation population in Guizhou Province. The participants were students ranging in age from the fifth grade of primary school to the third year of high school. A total of 600 questionnaires were distributed, 600 questionnaires were returned, and the recovery rate was 100%. Among them, 550 were valid questionnaires. Therefore, the effective rate of return was 91.7%. In this study, all subjects participated completely voluntarily. The responses of subjects were anonymous.

Among the participants, the ethnic representations were as follows: 134 Buyi, 146 Miao, 2 Bai, 1 Hui, 28 Yi, 189 Han, and 50 individuals from other minorities. Two hundred thirty-one were male, and 319 were female. The sample included 62 fifth, 74 sixth, 90 seventh graders, and 95 eighth, and 86 ninth-grade students. Forty-seven students were in the first, 49 students were in the second, and 47 students were in the third year of high school. The age range of all subjects was 13.88 ± 2.16.

### Measures

#### School adaptation scale for immigrant adolescents

We used a 15-item scale that is based on the psychological characteristics of immigrant adolescents. It uses a 5-point Likert scale for scoring. In the exploratory factor analysis of all items, the total variance explained rate was 66.771%, the KMO value was 0.881, and the spherical test was significant. Four factors were obtained and designated as follows: *academic adaptation* (including learning attitude, learning method, and others), four factors items; *teacher-student relationship adaptation* (including teachers' attitudes and behaviors toward students and students' attitudes and behaviors toward teachers), four items; *peer relationship adaptation* (including social skills and loneliness, and others), four items; and *new adaptation to family learning environment* (including the family learning environment after relocation, parents' attitude toward learning and others), three items. The factor loading size ranged from 0.569 to 0.825. Confirmatory factor analysis was carried out on the final four-factor structure of the 15 items yielding χ^2^/df = 1.928, NFI = 0.887, IFI = 0.942, TLI = 0.926, CFI = 0.941, RMSEA = 0.058. These results indicated that the model fit well. The internal consistency alpha coefficient of the scale was 0.867, showing that the school adaptation scale for immigrant adolescents had high validity and reliability.

#### General health inventory (GHQ)

The scale was compiled by Mäkikangas et al. (6) and revised by Zhang (46). It consists of 12 items, half of which are negative and half of which are positive. The scale is one-dimensional, using 4-point scoring. The higher the score attained, the more robust the individual's mental health. The internal consistency alpha coefficient of this scale for this study was 0.823.

#### Positive academic emotions questionnaire

The 30-item positive academic emotions subscale of the Adolescent Academic Emotions Scale was compiled by Yan and Guoliang (15). This subscale is divided into two dimensions: positive-high and positive-low arousal academic emotion. The subscale uses a 5-point Likert scale with higher scores indicating a more positive academic attitude. Its internal consistency alpha coefficient for this study was 0.927.

#### Behavioral tendency questionnaire for adolescent conduct problems

The questionnaire was compiled by Zhang et al. (47) and has a total of 14 items. Items are scored on a 5-point Likert scale. The questionnaire includes three dimensions: *violation tendencies* (six items), *addiction tendencies* (four items), and *aggressive tendencies* (four items). The higher the score, the stronger the tendency to manifest behavior problems. The internal consistency alpha coefficient of this scale for this study was 0.820.

### Analyses

All data were managed and statistically analyzed by SPSS and Amos (Versions 23.0). First, SPSS 23.0 was used to calculate the study variable descriptive statistics and their correlations. Next, we tested the mediating effect of positive academic emotions and conduct problems using PROCESS (Model 6 justified by 5,000 bootstraps). Finally, we used structural equation modeling (Amos 23.0) to investigate the fitness of the chain-mediating effect of positive academic attitude and conduct problems between school adaptation and mental health. The requirement of structural equation model for fitting exponent was χ2/df < 5, NFI > 0.8, IFI > 0.8, TLI > 0.8, CFI > 0.8, RMSEA < 0.08. Additionally, we used the percentile bootstrap method for mediation effect analysis.

### Procedure

The participants were recruited from schools in Guizhou Province of China. This study aimed at identifying possible predictors of the for the mental health of immigrant adolescents. Data collection was conducted in Guizhou Province of China and five schools were invited to participate which are in total. A detailed oral presentation was appeared to parents of adolescents attending the participating schools. All tasks were answered on paper. All measures in the study were administered electronically using EXCEL.

## Results

### Common method bias test

To avoid common method bias and control the quality of the survey, anonymity and reverse scoring were employed. According to Harman's single factor test, the exploratory factor obtained 19 factors with an eigenvalue >1 in the case of no pivot, and the explained variance of the first factor was 20.89%, far lower than the 40% critical standard proposed by Podsakoff et al. (48). This result confirmed the lack of serious common method bias in this study.

### Preliminary analysis

Independent samples *t*-test of variance was carried out to investigate differences in conduct problems by gender. Table 1 shows the results.

**Table 1:** *t*-test of immigrant adolescents' conduct problems.

**Variables**	**Gender**		***t***	***p***
	**Male (*n* = 231)**	**Female (*n* = 319)**		
Conduct problems	1.45 ± 0.48	1.30 ± 0.30	4.535^***^	0.000

*** *p* ≤ 0.001.

There were significant gender differences in conduct problems. Males exhibited far more conduct problems than girls.

### Correlation analysis

We calculated the means and standard deviations of school adaptation, mental health, positive academic emotions, and conduct problems among immigrant adolescents and performed a Pearson correlation analysis. Table 2 displays the results.

**Table 2:** Correlation analysis of school adaptation, mental health, positive academic emotions, and conduct problems among immigrant adolescents.

	**M**	**SD**	**Mental health**	**School adaptation**	**Positive academic emotions**	**Conduct problems**
Mental health	3.195	0.481	1			
School adaptation	3.297	0.728	0.534^***^	1		
Positive academic emotions	3.311	0.696	0.498^***^	0.667^***^	1	
Conduct problems	1.364	0.396	−0.464^***^	−0.327^***^	−0.321^***^	1

*** p ≤ 0.001.

There were significant positive correlations between school adaptation and mental health, school adaptation, and positive academic emotions. However, school adaptation was significantly negatively correlated with conduct problems. Mental health was significantly related to positive academic emotions. Mental health and conduct problems had a significant negative correlation. Finally, positive academic emotions were significantly negatively correlated with conduct problems.

### Mediation effect analysis

**Figure 2:**
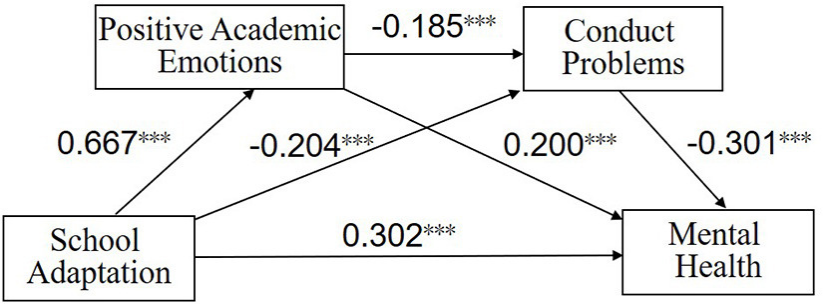
The chain mediating effect of positive academic emotions and conduct problems. ***p ≤ 0.001.

After controlling for gender and relocated adolescents' phase of studying, we performed a chain mediation regression analysis taking the school adaptation of the immigrant adolescents as the independent variable, mental health as the dependent variable, positive academic emotions and conduct problems as the mediating variables. We used MODEL6 in PROCESS and bootstrap repeated sampling 5,000 times. The results show that school adaptation significantly and positively predicted positive academic emotions (β = 0.667, *p* < 0.001). Furthermore, positive academic emotions (β = −0.185, *p* < 0.001) and school adaptation (β = −0.204, *p* < 0.01) significantly and negatively predicted conduct problems. Positive academic emotions (β = 0.200, *p* < 0.001), conduct problems (β = −0.301, *p* < 0.001), and school adaptation (β = 0.302, *p* < 0.001) together significantly predicted mental health. See Table 3, Figure 2 for details.

**Table 3:** The effect of school adaptation on mental health of immigrant adolescents: Chain mediation regression analysis.

**Outcome variable**	**Predictor variable**	***R***	***R* ^2^**	***F***	**β**	***t***
Gender					−0.0708	−2.0103^*^
Phase of studying					0.0170	0.7526
Positive academic emotions	School adaptation	0.667	0.445	439.469^***^	0.667	20.964^***^
Conduct problems	Positive academic emotions	0.355	0.126	39.437^***^	−0.185	−3.438^***^
	School adaptation				−0.204	−3.807^**^
Mental health	Positive academic emotions	0.633	0.401	121.774^***^	0.200	4.451^***^
	Conduct problems				−0.301	−8.495^***^
	School adaptation				0.302	6.710^***^

* p ≤ 0.05,

** p ≤ 0.01,

*** p ≤ 0.001.

Table 4 shows the results of the chain mediation analysis. The direct effect of school adaptation on mental health was 0.1998 (*t* = 6.710, *p* < 0.001, LLCI = 0.141, ULCI = 0.415), accounting for 56.6% of the total effect size. The total indirect effect size of positive academic emotions and conduct problems between school adaptation and mental health was 0.153, accounting for 43.4% of the total effect. The structural equation of the chain mediation model was established using Amos 23.0, χ2/df = 3.224, NFI = 0.949, IFI = 0.964, TLI = 0.949, CFI = 0.964, RMSEA = 0.064. It can be seen that the fitting index of the chain mediation model was relatively high. Therefore, school adaptation directly affected mental health and indirectly affected mental health through positive academic emotions and conduct problems. The indirect effects of specific school adaptation on mental health took three paths: the single mediating effect of positive academic emotions, the single mediating effect of conduct problems, and the chain mediating effect of positive academic emotions-conduct problems.

**Table 4:** Test of chain mediation effect of school adaptation for immigrant adolescents.

	**Indirect effect size**	**Boot standard error**	**BootCL Lower limit**	**BootCL upper limit**	**Relative mediation effect**
Total Indirect Effect	0.153	0.025	0.104	0.204	43.4%
School adaptation → Positive academic emotions → Mental health	0.088	0.024	0.041	0.136	25.0%
School adaptation → Conduct problems → Mental health	0.041	0.016	0.013	0.074	11.5%
School adaptation → Positive academic emotions → Conduct problems → Mental health	0.025	0.009	0.007	0.042	6.9%

Boot standard error, BootCL lower limit and BootCL upper limit refer to the standard error of the indirect effect estimated by the bias-corrected percentile bootstrap method (5,000 times), the lower limit and the upper limit of the 95% confidence interval, respectively.

## Discussion

This study found that male immigrant adolescents had significantly worse conduct problems than females, consistent with previous studies (49–52). The three problematic behavior tendencies of violation, addiction, and aggression are all external manifestations. In educational practice, males typically exhibit more conduct problems than females do. When adapting to a familiar environment, people often see that females are better-behaved, males are naughtier, and tend to annoy others more frequently. It is normal for young male immigrant adolescents to increase their nuisance behaviors and unexpected violations in an unfamiliar environment to which they have not yet adapted.

### The structure of the school adaptation scale for relocated adolescents

According to the characteristics of school adaptation for relocated adolescents, the factor structure and reliability of the relocated adolescent school adaptation scale are ideal. The internal consistency coefficient of the four dimensions of the relocated adolescent school adaptation scale was above 0.569, and the confirmatory factor analysis showed that the fitting index indicators of the relocated adolescent school adaptation scale were good. Specifically, the scale had a total of 15 items, including four items for academic adaptation, four items for teacher-student relationship adaptation, 4 items for peer relationship adaptation, and three items for new adaptation to family learning environment. However, test-retest reliability analysis and criterion analysis were not carried out in this study, and it was necessary to further explore the structure, reliability and validity of the scale in other groups.

### The relationship between school adaptation and mental health

This study found a significant positive correlation between school adaptation and mental health. Social-ecological system theory emphasizes that the developing individual is constantly growing and interacting with the surrounding environment. The impact of the environment on the development of children is multi-dimensional and varies (53, 54). After relocation, school and community culture affected the youth's growth. In addition, social policies affect the mental health of immigrant adolescents. This study found that school adaptation directly predicted mental health. This finding is consistent with previous studies (55), so we concluded that school adaptation is an essential factor affecting mental health.

### The mediating role of positive academic emotions and conduct problems

The urban relative poverty rate in many provinces and cities of the central and western regions is about 40% (56). Relative poverty has been shown to have an adverse effect on children's social-emotional development, and research has indicated that the adverse effect of relative poverty is bigger when children are older (57). The results of the current study show that mental health is affected by school adaptation, positive academic emotions, and conduct problem behaviors in relative poverty.

First, this study found that positive academic emotions played a mediating role between school adaptation and mental health. This result suggests that good school adjustment in adolescents has a positive impact on their mental health. The PPCT model (“Person-Process-Environment-Time” model) emphasizes that the systemic factors influencing individual development interact to form a network of forces. These forces blend to influence the individual's psychological development (53). Positive academic emotions in this study were a process factor influenced by school adaptation. The PPCT model proposes that positive emotions benefit individual development. Our results confirm the impact of positive academic emotions on mental health. Furthermore, they clarify the impact mechanism of school adaptation on mental health.

Second, this study found that conduct problems played a mediating role between school adjustment and mental health. This shows that adolescents with strong school adaptation were more likely to form good habits in relational, academic, and learning environments, were less likely to have conduct problems and were more likely to have better overall mental health. Conduct problems are not conducive to students' mental health. They are important indicators of bad behavior in students' development process (58). School adaptation may enhance mental health through the promotion of better behavior. Therefore, school adaptation can affect mental health indirectly by first reducing conduct problems.

Finally, this study found a chain-mediated mediating effect of positive academic emotions and conduct problems in school adaptation and mental health. On the one hand, positive academic emotions negatively predicted conduct problems. This study defined positive academic emotions as based on successful academic experiences. These experiences partly reflect good behavioral habits, which in turn improve conduct. Positive academic emotions facilitate a constructive psychological response mode. This response then influences the individual's ability to think about the environment, prompting young people to have a positive understanding of their character and enhancing mental health. Adolescents with adequate school adaptation develop positive academic attitudes. This disposition directly affects the individual's perception of adaptation and promotes high-quality behaviors. On the other hand, mental health is also a process in which adolescents interact with their own individual, various processes, and situational systems. Therefore, our research results showing that positive academic emotions and conduct problems had a chain-mediating effect on school adaptation and mental health further reveal the developmental mechanism of the psychological well-being of adolescent immigrants.

### Implications

It is necessary to strengthen inclusive mental health education for immigrant adolescents. When immigrant adolescents are enrolled in schools, special psychological files should be established for them. They should also be encouraged to deepen their understanding of academics, emotions, interpersonal relationships, and other areas through mental health education courses. This curriculum should help them to look at their learning constructively and form good study habits. At the same time, parents and teachers should provide praise and encouragement to promote the growth of their children. Sincere care and equal and friendly communication can help them reduce any sense of inferiority and alienation, and facilitate the establishment of their self-esteem and self-confidence, better integrating them into the collective and society.

It is also important to improve the comprehensive quality of all training offered to young people who have been relocated. Faced with changes in their living and educational environments, relocated youths are easily frustrated psychologically. They should receive more technological education, and education about integrating ethnically and culturally in the community. More activities featuring cultural practice should be offered. In addition, they should be encouraged to participate frequently in school and community affairs. At the same time, we should strengthen the cultivation and improvement of their self-esteem, stimulate their sense of future ownership, and help them to adjust to the school and community environments.

Giving relocated youth *fish* is not as effective as teaching them *how to fish*. Home-school co-governance, targeting the specific concerns of the school and the community, can be managed by recruiting volunteers and allowing parents and children to act as volunteers. These strategies will help to realize a virtuous circle of self-management and development and feelings of camaraderie and mutual assistance in many activities.

### Limitations

This study focused on the mental health of adolescent immigrants. We found that school adaptation enhanced youth mental health by promoting a positive academic attitude and reducing conduct problems. However, there are some research limitations to address. First, the self-report questionnaire method used cannot be used for robust inferences of causality. Second, the two mediating factors proposed in this study cannot be presumed to be the only ones from the perspective of theory and data. Therefore, in follow-up research, we should combine longitudinal research and other methods to continue to explore possible causal relationships. At the same time, we should further investigate other variables (e.g., self-esteem, resilience, and conflict adaptation) affecting the mental health of immigrant adolescents to define a more reasonable impact mechanism. Finally, the generalization of the study results requires further support from future studies.

## Conclusion

This study yielded important findings on how school adaptation relates to mental health among adolescent immigrants, requiring replication, extension, and further exploration. This study's results suggest that academic attitude and conduct problems are underlying mechanisms through which school adjustment is associated with mental health. In addition, it reveals a chain mediation relationship between positive academic emotions and conduct problems with school adaptation and mental health. Therefore, experimental and longitudinal designs can be employed in future studies to infer causal relationships between variables. In relocation situations, there should be a focus on the impact of relocation on adolescents' adaptation on their mental health.

## Data availability statement

The raw data supporting the conclusions of this article will be made available by the authors, without undue reservation.

## Ethics statement

The studies involving human participants were reviewed and approved by the Research Ethics Committee of the School of Education Science, Xingyi Normal University for Nationalities. Written informed consent to participate in this study was provided by the participants' legal guardian/next of kin.

## Author contributions

LX: conceptualization, investigation, writing–original draft, visualization, and revised manuscript. WZ: conceptualization, methodology, investigation, statistical analysis, data curation, and visualization. HW: project administration and funding acquisition. All authors read and approved the final manuscript.
